# An Improved Experimental Method for Simulating Erosion Processes by Concentrated Channel Flow

**DOI:** 10.1371/journal.pone.0099660

**Published:** 2014-06-20

**Authors:** Xiao-Yan Chen, Yu Zhao, Bin Mo, Hong-Xing Mi

**Affiliations:** 1 College of Resources and Environment/Key Laboratory of Eco-environment in Three Gorges Region (Ministry of Education), Southwest University, Chongqing, China; 2 State Key Laboratory of Soil Erosion and Dryland Farming on the Loess Plateau, Institute of Soil and Water Conservation, CAS and MWR, Yangling, China; DOE Pacific Northwest National Laboratory, United States of America

## Abstract

Rill erosion is an important process that occurs on hill slopes, including sloped farmland. Laboratory simulations have been vital to understanding rill erosion. Previous experiments obtained sediment yields using rills of various lengths to get the sedimentation process, which disrupted the continuity of the rill erosion process and was time-consuming. In this study, an improved experimental method was used to measure the rill erosion processes by concentrated channel flow. By using this method, a laboratory platform, 12 m long and 3 m wide, was used to construct rills of 0.1 m wide and 12 m long for experiments under five slope gradients (5, 10, 15, 20, and 25 degrees) and three flow rates (2, 4, and 8 L min^−1^). Sediment laden water was simultaneously sampled along the rill at locations 0.5 m, 1 m, 2 m, 3 m, 4 m, 5 m, 6 m, 7 m, 8 m, 10 m, and 12 m from the water inlet to determine the sediment concentration distribution. The rill erosion process measured by the method used in this study and that by previous experimental methods are approximately the same. The experimental data indicated that sediment concentrations increase with slope gradient and flow rate, which highlights the hydraulic impact on rill erosion. Sediment concentration increased rapidly at the initial section of the rill, and the rate of increase in sediment concentration reduced with the rill length. Overall, both experimental methods are feasible and applicable. However, the method proposed in this study is more efficient and easier to operate. This improved method will be useful in related research.

## Introduction

Soil erosion is a serious environmental problem that threatens agricultural safety and sustainable development due to land degradation [1]–[5]. As an important component of hill slope soil erosion, rill erosion is especially dangerous on cultivated slopes and upland areas. Eroding rills are formed by concentrated surface runoff and function as sediment source areas and sediment transport vehicles [6]–[9]. Considering its importance, the mechanism of rill erosion has long been the focus of study.

As early as 1981, Foster et al. [10] differentiated upland soil erosion into inter-rill erosion and rill erosion. They indicated that rill erosion contributed more significantly to sediment production than inter-rill erosion. Since then, many researchers have studied rill morphology, the hydraulics of rill flow, and the rill erosion process. The results indicate that the parameters, such as sediment concentration, soil detachment rate, sediment transport capacity, soil erodibility and critical shear stress, can be used to characterize the rill erosion process. Sediment concentration and soil detachment rate are the most relevant indicators of erosion. Sediment transport capacity expresses the potential sediment entrainment ability of the concentrated flow in rills. Soil erodibility and critical shear stress provide the criteria for determining the occurrence and quantity of rill erosion [11]–[17].

In recent decades, various research techniques have been applied to study rill erosion. However, information regarding spatially distributed rill erosion is limited. Most existing rill erosion data are spatially integrated, as measured at the rill outlet [18]–[21]. Spatially integrated rill erosion data do not adequately describe the dynamics of the rill erosion process.

Traditional measurement methods cannot easily quantify the rill erosion process due to the complexity of quantifying erosion among various rill segments. Consequently, new methods that provide dynamic rill erosion process are needed.

Recently, the rare earth element (REE) was utilized to trace the temporal and spatial distribution data of rill erosion [22]–[24]. The method successfully quantified the rill erosion process. However, the REE method is not economical or efficient, and need special and expensive facilities to conduct the measurement. Lei et al. [13] suggested an experimental method for studying rill erosion process. In their method, the sediment concentrations produced from rills of different lengths are measured. Next, the measured sediment data are integrated to produce a spatially distributed rill erosion process. However, the approach was determined to be time consuming and disruptive to the continuity of the rill erosion process. Aksoy et al. [16], [17] performed experimental analysis in a laboratory flume under simulated rainfall by pre-formed rill. They used the experimental data to relate sediment concentration to slope gradient and rainfall intensity.

Rill erosion involves such processes as infiltration of rill, winding of flow path and failure of side walls due to randomized scouring and deposition. These processes are interactive with the most important rill erosion components such as detachment rate, transport capacity, shear stress and sediment concentration along the rill to be computed or estimated.

Along a width-fluctuated rill, the sediment concentrations are much lower than those along a well-defined rill with constant water flow [25]. Naturally developed rills in laboratory experiments involve periodic change of detachment and deposition responsible for rill width fluctuation, widening where deposition occurs and narrowing when scouring takes place [26]. Randomized side wall failure during rill erosion process does cause underestimation of sediment concentration at locality. Still the underestimated sediment load in the water flow is much higher than that after deposition occurrence. Furthermore, water flow of lower sediment concentration causes higher scouring of rill bed to contribute more sediments to compensate the water flow [27], [28]:
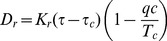
(1)where, *D_r_* (kg m^−2^ s) is the contribution of rill detachment rate to rill sediment load, *K_r_* (s^−1^) is the erodibility of the soil; *τ* (kg m^−2^) is the shear stress of flowing water, *τ_c_*(kg m^−2^) is the critical shear strength of soil; *c* (kg m^−1^) is the sediment concentration and *q* (m^2^ s^−1^) is the flow rate of a unit width, *T_c_* (kg s^−1^ m) is the sediment transport capacity of the water flow [28].

Therefore, it seems rational to use well-defined rill for quantitative study of rill erosion.

The objectives of this study were to: 1) develop an improved experimental method for determining the rill erosion process; 2) determine the rill erosion process with experimental data under various hydraulic conditions; 3) assess the sediment concentration and erosion process with previously reported experimental data.

## Methods and Materials

In regular rill erosion, the rill width changes periodically due to the erosion and deposition. And the sediment concentration could be much lower than that in well-defined rill under constant water flow. In order to overcome this problem of rill width fluctuation, well-defined rill was constructed in this experiment.

**Figure 1:**
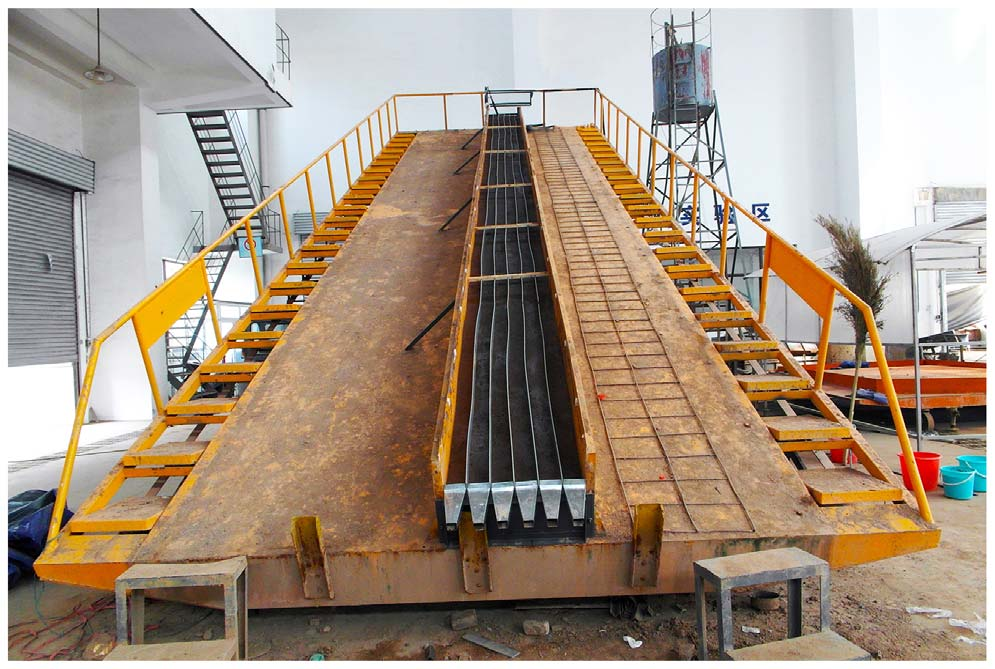
The experimental setup. A picture shows the experimental setup.

The experiments were conducted in the State Key Laboratory of Soil Erosion and Dryland Farming on the Loess Plateau, Institute of Soil and Water Conservation, Chinese Academy of Science and Ministry of Water Resources, Yangling, Shanxi Province, China. A platform that was 12 m long and 3 m wide was used as a base to construct a flume that was 12 m long, 0.6 m wide and 0.5 m deep. The flume was sub-divided into six rills that were 0.1 m wide and 12 m long using upright PVC boards to form well-defined rills (Fig. 1). The PVC board surfaces were glued with the experimental soil particles on both sides to create a roughness equivalent to the soil surface so as to minimize the boundary effect on the hydraulic and erosion processes, in case when water flow becomes contact with the boards.

The experimental soil materials were collected from the Ansai Research Station of Soil and Water Conservation on the Chinese Loess Plateau. The soil contains 15.92% clay (<0.005 mm), 63.90% silt (0.005 to 0.05 mm), and 20.18% sand (>0.05 mm), which is classified as a silt-loamy soil. The soil was air dried before being crushed and passed through an 8 mm square sieve.

The bottom 5 cm of the flume was paved with a clay loamy soil (24.9% sand particles, 43.4% silt particles and 31.8% clay particles) to achieve a bulk density of approximately 1.5 g cm^−3^ (i.e., an imitation of the plow pan layer). On top of the plow pan layer, the experimental soil was packed in 5 cm thick layers to a total depth of 20 cm, at the bulk density of approximately 1.2 g cm^−3^. The soil near the PVC boards was packed slightly higher than the middle in aim to converge the water flow to the center of the rill and to minimize the boundary effect as much as possible. The prepared rills were saturated with the rain-simulator and allowed to drain for 24 h to ensure an even and homogeneous initial soil moisture profile in an experimental run, and among different experimental runs.

A water tank was used to supply the water flow at the designed discharge rate. An additional specially designed device was used at the rill flow inlet to accelerate the water flow to a velocity level close to the rill flow. Gauze cloth approximately 0.2 m in length was placed at soil surface of the rill inlet to protect the rill surface from being directly scoured by the water flow.

Experimental runs were conducted for five slope gradients (5°, 10°, 15°, 20° and 25°) and three flow rates (2 L min^−1^, 4 L min^−1^, and 8 L min^−1^) with 3 replicates.

Water flow at the designed rate was introduced into the rill at the top end after the flume was adjusted to the designed slope gradient. In Lei et al.'s [13], [29] experiments, sediment-laden water samples were collected at outlets of rill of various lengths before the collected data were integrated to produce the sediment process of the entire 8 m rill. Here, after steady water flow in the rill was established, sediment-laden water samples were simultaneously taken along the rill at distances of 0.5 m, 1.0 m, 2.0 m, 3.0 m, 4.0 m, 5.0 m, 6.0 m, 7.0 m, 8.0 m, 10.0 m, and 12.0 m from the water flow inlet, which is an improvement on the previous method. Thus, the sediment delivery process was determined along the rill. Three samples were taken at 1.0-minute interval for each experimental run. Therefore each experiment lasted less than 5 minute after steady flow was established. The average value of the three samples at each location was used to determine the sediment concentration at various rill lengths. The sediment concentrations that were measured at the various locations formed the sediment delivery process along the rill.

## Results and Discussion

### Sediment concentration along the rill

**Figure 2:**
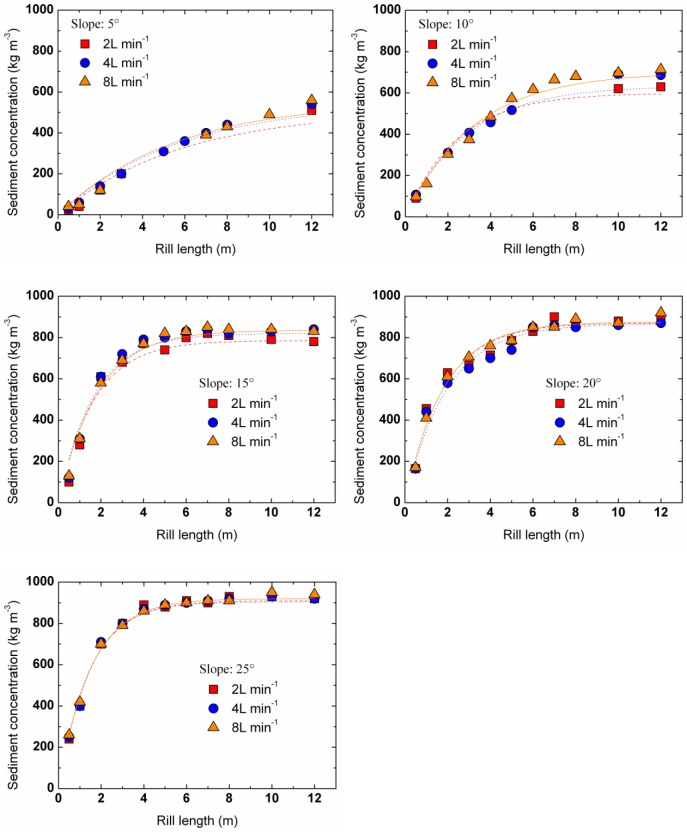
Sediment concentration along the rill at various slope gradients. The variation trend of the measured sediment concentrations along the rill under three flow rates and five slope gradients. The sediment concentration increased exponentially with the rill length for all of the flow rates and slope gradients. The sediment concentration also increased with the slope gradient and flow rate at lower slope gradients (5°, 10° and 15°).

The measured sediment concentrations along the rills under various flow rates and slope gradients are presented in Fig. 2. The experimental data exhibited a well defined trend between the sediment concentration and rill length, which can be described by:
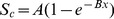
(2)where *S_c_* (kg m^−3^) is the sediment concentration, *x* (m) is the rill length, and *A* (kg m^−3^) and *B* (m^−1^) are regression coefficients, with *A* as the maximum possible sediment concentration and *B* as the attenuation coefficient to indicate how fast of the reduction in sediment increase.

The experimental data presented in Fig. 2 were regressed with Eq. (2). The regression parameters are listed in Table 1. The regression results indicated that the sediment concentrations for all the experimental runs increased exponentially with the rill length. Polyakov and Nearing [30] and Lei et al. [31] studied the sediment concentrations in rills and concluded that the relationship between sediment concentration and rill length is well-described by Eq. (1) too. The sediment concentration increased rapidly at the initial section of the rill, where the water contains limited amount of sediments. The increase in sediment concentration reduced rapidly along the rill. These sediment concentration distributions were more evident at steeper slopes. It is possible that clear water (no sediment particles) that is introduced into the rill at the inlet spends limited energy to transport sediments and high energy to detach soil at the upper part of the rill. According to the Water Erosion Prediction Project (WEPP) model [28], clear water has a highest detachment potential. The increase in sediment concentration decreases to zero when the water is saturated with sediments (up to its transport capacity). Almost all the energy of the water flow is used for sediment transportation [32].

**Table 1:** Regression parameters of Eq. (1).

Slope (°)	flow rate (L min^−1^)	*A*(kg m^−3^)	*B* (m^−1^)	*R^2^*	*F*	*Prob>F*
5	2	510	0.17	0.95	172.74	5.09E-08
	4	550	0.18	0.97	1174.60	8.76E-08
	8	560	0.18	0.97	593.85	2.17E-06
10	2	625	0.41	0.99	622.72	7.85E-10
	4	635	0.35	0.96	1020.01	1.57E-13
	8	675	0.31	0.98	3072.75	1.48E-13
15	2	785	0.60	0.95	1846.31	1.99E-08
	4	820	0.60	0.97	2868.62	5.75E-09
	8	835	0.56	0.98	4788.88	1.80E-09
20	2	870	0.56	0.96	2906.29	8.80E-10
	4	865	0.52	0.96	3088.36	9.55E-10
	8	875	0.56	0.98	7395.75	7.12E-11
25	2	910	0.68	0.99	11025.60	1.35E-09
	4	905	0.69	0.99	11587.59	6.75E-10
	8	910	0.68	0.99	16567.57	8.57E-11

In Table 1 all the coefficients of determination (*R^2^*) were higher than 0.95, which indicate that Eq. (2) fits well the sediment concentration along the rill. All the values of (*Prob>F*) from the *F-test* were rather low (i.e., less than 8.76 E^−8^), to indicate that the results were statistically significant (*F-test* with *α* = 0.01).

As indicated in Fig. 2, all the sediment concentrations increased with the rill length and eventually reached the maximum value of *A*. The value of *A* was the sediment concentration when the water flow was saturated with sediment particles, which represents the sediment concentration at the transport capacity of the water flow. Parameter *B* was a reduction coefficient that indicates the attenuation of the increase in sediment concentration. From Table 1, the value of *A* increases with the slope and ranges from 510 to 910. Thus, the sediment transport capacity increased rapidly with the slope gradient. *A* did not vary significantly with the flow rate under the same slope gradient. The value of *A* changed slightly between 20° and 25°, which indicates that a critical slope gradient between 20° and 25° may exist.

In summary, the sediment concentration for sediment saturated flow represents the transport capacity; thus, the sediment transport capacity and the rill length can be estimated.

### Relationship between sediment concentration, slope and flow rate

The sediment concentration increased with the slope and flow rate at lower slope gradients (5°, 10° and 15°). This is consistent with the findings of other researchers [33], [34]. For rill erosion, the inflow rate and slope gradient are the most influential factors. Steeper slopes and higher flow rates provided greater flow shear force. These factors enhance soil erosion by either increasing the soil detachment rate or by weakening the protective power of the soil surface to resist erosion [35]–[37]. Furthermore, the curve defined by Eq. (2) fits well with the sediment concentration under the three flow rates. The sediment concentrations increased with the slope gradient, but they only increased slightly with the flow rate (Fig. 2). For steep slope gradients (20° and 25°) the sediment curves under three flow rates nearly overlapped at the same slope gradient. The slope may have a greater influence on the sediment concentration than the flow rate. Although the sediment concentrations were approximately the same, the higher flow rate delivered proportionally more sediment (proportional to the flow rate).

To better understand and clarify the relationship between sediment concentration, slope gradient and flow rate, several functions were tried to fit the relationship between the sediment concentration at transport capacity, slopes and flow rates. The following function was found a simple and appropriate fit for the experimental data:
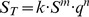
(3)where *S_T_* (kg m^−3^) is the sediment concentration at transport capacity, *S* is the slope gradient (%), *q* is the flow rate (L min^−1^) and *m* and *n* are regression coefficients. The regression results are listed in Table 2.

**Table 2:** Regression coefficients under various slope gradients and flow rates.

Slope (°)	flow rate (L min^−1^)	*K*	*m*	*n*	*R^2^*	*Prob>F*
5	2	0.83	2.45 a	1.64 a	0.95	4.59E-04
	4	0.89	2.23 b	1.13 b	0.92	4.34E-04
	8	0.87	1.99 c	1.03 bc	0.95	9.51E-04
10	2	0.80	2.12 bc	0.96 bc	0.86	7.33E-04
	4	0.74	1.94 c	0.91 c	0.89	3.11E-03
	8	0.78	1.87 cd	0.70 cd	0.88	3.64E-03
15	2	0.86	1.96 c	0.61 d	0.93	5.74E-04
	4	0.79	1.89 cd	0.57 de	0.88	1.84E-03
	8	0.83	1.76 d	0.55 de	0.84	1.15E-02
20	2	0.82	1.87 cd	0.54 de	0.95	9.73E-04
	4	0.71	1.83 cd	0.49 de	0.93	7.69E-03
	8	0.75	1.75 d	0.38 e	0.93	7.40E-03
25	2	0.78	1.79 d	0.41 de	0.95	1.20E-03
	4	0.69	1.76 d	0.39 e	0.93	7.04E-03
	8	0.74	1.69 d	0.38 e	0.90	1.67E-02

Footnote: Significant differences between the values are indicated by letters (*p<0.05*).

Regression analysis indicated that *m* decreased steadily with slope gradient slope and ranged from 2.45 to 1.69. This indicates that the increase in sediment concentration at transport capacity decreases with the slope gradient. However, *n*, decreased with slope gradient and flow rate, indicating that the increase in sediment concentration declines with the flow rate. All of the coefficients of determination (*R^2^*) were higher than 0.84, which indicates that Eq. (3) is able to quantify the sediment concentration at transport capacity of rill erosion. Both the regression coefficients in Eq. (3), *m* and *n*, were very significant (*F-test* with *α* = 0.01), which illustrates that the slope and flow rate were both critical parameters that influence sediment concentration at transport capacity of rill erosion process. Generally, sediment concentration at transport capacity increased with the slope gradient and flow rate (Table 2). Additionally, the values of *m* were all greater than *n*; the sediment concentration at the transport capacity increased nearly quadratically (*m*≈2) with the slope gradient, but increased less than linearly (*n*<1) with the flow rate. This indicates that the slope is much more important than the flow rate with respect to the sediment transportation [38]–[40]. Lei et al. [31] suggested that the sediment concentration at transport capacity increased quadratically with slope gradient and exhibited an approximately linear increase with flow rate, which supported our results. Similarly, Aksoy et al. [17] concluded that slope created a linear increase in sediment concentration but the flow rate had practically no effect on sediment concentration.

According to Table 2, the *m* values of 5°, 10°, 15° and 20° and 25° were significantly different from each other. However, *m* did not display statistically significant differences between 20° and 25°. Based on the given slope and flow rate, the sediment concentration at the transport capacity increased more significantly with the slope gradients than with flow rates from 5° to 20°. However, when the slope gradient increased from 20° to 25°, the sediment concentration was more stable. The *n* values revealed similar results: the values at 20° and 25° were obviously different from those at the other slopes, but significant differences did not exist between the two slope gradients. That is to say, the sediment concentration had almost achieved its maximum value (i.e., a certain slope gradient between 20° and 25° was the critical erosion point of the experimental soil).

### Comparison of rill erosion processes measured by various methods

**Figure 3:**
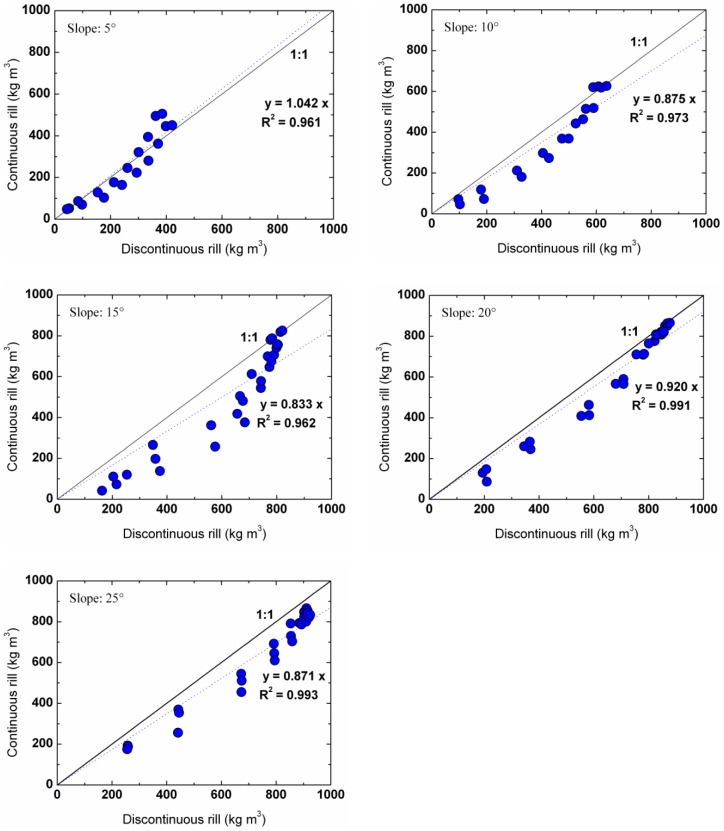
The sediment concentrations measured from continuous rills in this study and discontinuous rills in Lei et al. (2002). The sediment concentrations measured from continuous rills in this study and discontinuous rills in Lei et al. (2002) which used a similar methodology. The relative error produce by these two methods is less than 15%, which suggests that both of the methods are feasible.

In the experiments by Lei et al. [29], the soil type, designed slope gradients and flow rates were all identical to those of the present study. However, the rill length used by the previous experiment was 8.0 m, whereas the rill length used in the present study was 12.0 m. Furthermore, in the previous study, the sediment concentration process along the 8 m rill was integrated from experimental data of rills of various lengths (i.e., 0.5 m, 1 m, 2 m, etc, up to 8 m), slope gradients and flow rates. In this study, simultaneous and continuous sampling procedures were used. To compare the two data sets, only the data at the upper 8.0 m in this experiment were used (Fig. 3), with the experimental data of discontinuous rill as X-axis and the data of this study as Y-axis. The closer the dots in Fig. 3 are to the 1∶1 line, the better the agreement of the two data sets. The data sets indicate that the sediment concentrations from the present experimental method were approximately 1.04, 0.88, 0.83, 0.92, and 0.87 times of the previous experimental data sets at the slope gradients of 5°, 10°, 15°, 20° and 25°, respectively. The coefficients of determination (*R^2^*) were all greater than 0.96, which indicates that the two datasets were approximately the same and closely correlated.

The previous experimental technique produced reasonable rill erosion data, but it produced slightly higher sediment concentrations. This could have been caused by random errors in sampling, experiment preparation, etc. The method suggested in this study is more efficient. Simultaneous sampling along the rill is more rational. The relative error produced by these two methods is less than 15%, which suggests that both methods are feasible and applicable. The improved method suggested in this study can measure the rill erosion simultaneously and continuously.

## Conclusions

Given the limits of the method used in previous rill erosion research, an improved and easy to use method of studying rill erosion processes by means of simultaneous sampling of sediment-laden water was suggested. The dynamic changes and distributions of sediment concentrations along the rill were measured for a silty-loam soil over a range of slope gradients and flow rates. The results indicate that sediment concentration increased exponentially with the rill length for all of the flow rates and slope gradients. The sediment concentration also increased with the slope gradient. The data proved that the slope gradient and flow rate are both important parameters for rill erosion, but the slope gradient seemed to be more influential on the sediment concentration. The data computed from the improved method were compared with the data from a previous study, which suggested that this study is a feasible mean to simulate rill erosion process. However, this improved experimental method needs further examination at field scale and more attention should be paid on measures to minimize the boundary effect.
